# Effects of Multiwall Carbon Nanotubes on Premature Kidney Aging: Biochemical and Histological Analysis

**DOI:** 10.3390/toxics11040373

**Published:** 2023-04-14

**Authors:** Ji-Eun Kim, Myung-Haing Cho

**Affiliations:** 1 Laboratory of Toxicology, College of Veterinary Medicine, Seoul National University, Seoul 151-742, Republic of Korea; 2 Program in Developmental & Stem Cell Biology, The Hospital for Sick Children, Toronto, ON M5G 0A4, Canada; 3 RNABIO, Seongnam 13201, Republic of Korea

**Keywords:** multiwall carbon nanotubes (MWCNTs), kidney, toxicity, premature aging, multiwall carbon nanotubes

## Abstract

Carbon nanotubes (CNTs) have gained much attention due to their superb properties, which make them promising options for the reinforcing composite materials with desirable mechanical properties. However, little is known about the linkage between lung exposure to nanomaterials and kidney disease. In this study, we compared the effects on the kidneys and aging for two different types of multiwall carbon nanotubes (MWCNTs): pristine MWCNTs (PMWCNTs) and acid-treated MWCNTs (TMWCNTs), with TMWCNTs being the preferred form for use as a composite material due to its superior dispersion properties. We used tracheal instillation and maximum tolerated dose (MTD) for both types of CNTs. MTD was determined as a 10% weight loss dose in a 3-month subchronic study, and the appropriate dosage for 1-year exposure was 0.1 mg/mouse. Serum and kidney samples were analyzed using ELISA, Western blot, and immunohistochemistry after 6 months and 1 year of treatment. PMWCNT-administered mice showed the activation of pathways for inflammation, apoptosis, and insufficient autophagy, as well as decreased serum Klotho levels and increased serum levels of DKK-1, FGF-23, and sclerostin, while TMWCNTs did not. Our study suggests that lung exposure to PMWCNTs can induce premature kidney aging and highlights a possible toxic effect of using MWCNTs on the kidneys in the industrial field, further highlighting that dispersibility can affect the toxicity of the nanotubes.

## 1. Introduction

Multi-walled carbon nanotubes (MWCNTs) have unique mechanical, electrical, and thermal properties. With their potential for massive adoption in the industrial field, concerns over unanticipated effects have been emphasized [1]. Exposure to MWCNTs has been associated with various inflammatory responses in different organs. For instance, MWCNTs exposed to the lungs have been shown to cause granulomatous inflammation [2], while exposure in the liver has been linked to nonalcoholic steatohepatitis [3], and neuroinflammation has been observed in the brain [4]. In addition, MWCNT exposure on the skin has also been reported to induce inflammation [5]. Despite the fact that the kidney is a toxicologically vulnerable organ and plays an important role in body hormone and mineral homeostasis [6], toxicity evaluations for lung exposed MWCNTs in the kidney have not been reported. In particular, the kidney is a typical casualty of aging, as shown by the higher prevalence of chronic kidney disease (CKD) in people older than 70 [7] and the higher susceptibility of acute kidney injury (AKI) in elderly people [8]. This is because kidney cells barely express the enzyme telomerase [9], resulting in a limited ability to recover after acute injuries [10].

Premature aging is a process associated with a progressive accumulation of deleterious changes over time, an impairment of physiological functions, and an increase in the risk of diseases and death [11,12,13]. Regardless of genetic background, aging can be promoted by the lifestyles and environmental conditions to which our genes are exposed [12,14]. The kidneys play a key role in the regulation of body homeostasis by eliminating toxic substances and waste products, or maintaining acid-base balance and producing hormones such as calcitrol and erythropoietin [15]. Chronic kidney disease is a well-known condition that accelerates cellular senescence and premature aging through toxic alterations in the internal environment [12,16,17]. This occurs through several mechanisms, including increased reactive oxygen species generation [18], DNA and mitochondria damage [19], persistent inflammation [20], phosphate toxicity [11,21], stem cell depletion [22], decreased Klotho expression [11,23] and telomere shortening [10]. Through these mechanisms, patients with chronic kidney disease show premature aging phenotypes that are very similar to anti-aging protein Klotho knock-out mice such as hyperphosphatemia, vascular calcification and decreased mineral bone density [12,24]. These similarities led to the theory that CKD is a premature-aging-like syndrome caused by hyperphosphatemia [12]. Patients with CKD show increased oxidative DNA damage and shortened telomerase, supporting the theory.

Kidney premature aging is a complex process characterized by pathological and functional changes that accumulate over the course of a lifetime [25]. The main structural and functional changes in the aging kidney include glomerular, tubulo-interstitial, vascular, and endocrine changes [26]. As for the underlying molecular mechanisms, increased apoptosis is an intriguing theory for the increased cell damage observed with aging, as it represents a convergence between increased acute kidney injury (AKI) in the aging kidney and chronic renal dysfunction due to tubular loss [26]. In addition to this, autophagy is also reported to be altered in the aging kidney. Cui et al. reported that autophagy was not induced during ischemic stress in tubular cells in the aging kidney [27]. The increased chronic inflammation, which is characterized by the accumulation of macrophages and lymphocytes in the renal interstitium, also promotes renal senescence [13].

Based on the current state of MWCNT study and application, we used two different kinds of MWCNTs for the comparison of kidney toxicity and the effects on premature aging: pristine multi-walled carbon nanotubes (PMWCNTs) and acid treated multi-walled carbon nanotubes (TMWCNTs). The PMWCNTs were raw MWCNTs as purchased, and the TMWCNTs were highly dispersed PMWCNTs subjected to a multistep acid treatment using both hydrochloric and nitric acids [2]. As the number of CNT researchers and workers that use highly dispersible functionalized MWCNTs are increasing, we used TMWCNTs as the test materials [28]. In a previous study, we showed that TMWCNTs clear faster than PMWCNTs [2], and that PMWCNTs can induce non-alcoholic steatosis hepatitis-like phenotypes [3]. Although inhalation is the most ideal method for testing airborne materials, the equipment is expensive to acquire and maintain and it is hard to generate and characterize exposure from the atmosphere especially with fibrous MWCNTs. Moreover, anatomically, mice have a longer, more complex, and smaller proximal nasal airway path than humans, which provides better protection to the lower respiratory tract by improving filtration, absorption, and disposal of airborne particles and gases. In contrast, humans have simple middle and inferior turbinates in their nose [29]. Consequently, the inhalation of MWCNTs in mice may not achieve the intended delivery of particles to the lungs due to these structural differences. Therefore, direct instillation of the test material into the lungs was employed as an alternative to inhalation in this study. Tracheal instillation also has certain advantages over inhalation such as having a qualitatively reliable method when comparing different materials as the actual dose delivered to the lungs of mice can be defined accurately [30]. Based on these advantages, we compared the kidney damage and premature aging effect of PMWCNTs and TMWCNTs in the murine lung at 6 months and 1 year post tracheal instillation. This study has found that exposure of the lungs to PMWCNTs can induce premature kidney aging, indicating a possible toxic effect on the kidneys from the use of MWCNTs in industrial settings. This highlights the importance of considering the dispersibility of MWCNTs, as it can affect their toxicity.

## 2. Materials and Methods

### 2.1. Preparation of Multi-Walled Carbon Nanotubes

MWCNTs CM-95^TM^ were purchased from Hanhwa Nanotech (Seoul, Republic of Korea), and the diameter and purity were reported as 12.5 ± 2.5 nm and >95%, respectively, by the manufacturer. MWCNTs not treated with anything were termed pristine-MWCNTs (PMWCNTs) and acid-treated MWCNTs were termed treated-MWCNT (TMWCNTs). These were prepared as previously reported [2,3] Briefly, PMWCNTs were mixed with HNO_3_ and H_2_SO_4_ (1:3 = *v*/*v*); approximately 10 mL of HNO_3_ and 30 mL of H_2_SO_4_ for 1 g of PMWCNTs. Then, the mixture was sonicated during 25 min for assisting the dispersal of MWCNTs. These mixtures were then refluxed at 120 °C for 90 min, and washed with deionized water and filtered using pore size of 0.8 µm, DM Metricel 800 (Pall Life Science, Port Washington, NY, USA). MWCNTs were weighed in a 10 mL glass vial in the fume hood and then was dry-heat sterilized at 200 °C for 1 h. They were suspended in sterile saline at a final concentration of 1 mg/mL in 10 mL glass vials and were sonicated for 15 min in a water sonicator bath (5510-DTH; Branson, Danbury, CT, USA); it was used as the stock solution for tracheal instillation. To prevent physical changes of MWCNTs, the stock solution of each MWCNT was prepared immediately before each cell experiment.

### 2.2. Transmission Electron Microscopy (TEM)

A drop of the MWCNTs solution was placed on a formvar/carbon-film-coated 400 mesh Cu TEM grid (Samchang Commercial Co., Ltd., Seoul, Republic of Korea), followed by sonicating in ethanol for 3 min. Morphology of MWCNTs were analyzed using energy-filtering TEM (EF-TEM) on a LIBRA 120 instrument (Carl Zeiss, Oberkochen, Germany) with voltage of 120 kV.

### 2.3. Raman Spectroscopy

Using a confocal microscope Raman system (LabRAM 300; JY-Horiba, Edison, NJ, USA) equipped with an optical microscope (Olympus, Tokyo, Japan), Raman scattering signals were collected in a 180° back-scattering geometry following the detection by a spectrometer equipped with a thermoelectrically cooled (−70 °C) charge-coupled device (CCD) detector. Power of the laser was approximately 1.2 mW at the samples, and the excitation source was a 647-nm Kr laser (Innova I-301; Coherent, CA, USA). Raman signals were collected on the selected point for 1 s.

### 2.4. Inductively Coupled Plasma-Atomic Emission Spectroscopy (ICP-AES)

ICP-AES was performed using an Optima-4300 DV (PerkinElmer, Waltham, MA, USA). In the mixture of 5 mL of aqua regia, 30 mg of MWCNTs were dissolved and mixed with 2 mL of hydrofluoric acid for 5 h in 180 °C. Then, the mixture was centrifuged and separate the residual solids. Lastly, the concentrations of metal elements in aqua regia were analyzed after dilution with distilled water to 20 mL.

### 2.5. Dosage of PMWCNTs and TMWCNTs

To determine the dosage for 1 year of exposure to MWCNTs, we used data from our previous 3-month subchronic exposure experiment [2]. Over 3 months, mice were treated with 0.1 mg PMWCNTs and TMWCNTs, and the dosage was determined according to the dose that was approximately less than 10% of the body weight lost compared to the control group (Figure 2B).

### 2.6. Animal Experiments and Tracheal Instillation

Six-week-old male C57BL/6 mice were purchased from ORIENT BIO (Seongnam, Republic of Korea) and quarantined one week prior to the experiment. The mice were kept in the laboratory animal facility with temperature and relative humidity maintained at 23 ± 2 °C and 50 ± 20%, respectively, under a 12-h light/dark cycle, and given food and water ad libitum. All methods used in this study were approved by the Animal Care and Use Committee at Seoul National University. A total of 50 µL of PMWCNT and TMWCNT suspensions were administered via intratracheal instillation to the mice in doses of 10 or 100 µg/mouse. After being anesthetized intra-peritoneally with ketamine/xylazine (150/15 mg/kg mixture) solution, a 24-gauge catheter was intubated into the trachea. Then, 50 µL of the MWCNT suspension was instilled into the catheter one drop at a time. Vehicle control mice received 50 µL of sterilized saline. The animals were sacrificed at the time points of 6 months and 1 year following injection (n = 5/group).

### 2.7. Hematoxylin and Eosin (H&E) and Masson’s Trichrome Staining

The kidneys were decapsulated and fixed in 10% neutral buffered formaldehyde overnight. The tissues were then paraffin processed, sectioned at a thickness of 3 μm, and transferred to a Plus slide (Fisher Scientific, Pittsburgh, PA, USA). For histologic analysis, the tissue sections were deparaffinized in xylene, rehydrated and stained with H&E (Sigma-Aldrich, Saint Quentin Fallavier, France), and Masson’s Trichrome staining (HT15, Sigma-Aldrich) according to the manufacturer’s protocol.

### 2.8. Kidney Histology

Scoring of tubular degeneration and mononuclear/lymphocytic infiltrates in the kidneys was performed in a blinded fashion by veterinary pathologists based on a previously described method [31].

### 2.9. Western Blot Analysis

Kidneys were homogenized using lysis buffer, and protein concentrations were measured with a Bradford solution (Bio-Rad, Hercules, CA, USA). Western blot analysis was performed as described previously (Kim et al., 2014 [3]). Anti-nuclear factor kappa-light-chain-enhancer of activated B cells (NF-κB) p65 (sc-372), BAD (sc-943) and BAX (sc-526) were purchased from Santa Cruz Biotechnology, Inc. (Santa Cruz, CA, USA). Anti-alpha-smooth muscle actin (α-SMA) (ab5694), anti-F4/80 (ab6640), anti-Klotho (ab18131), and anti-p62 (SQSTM1) (ab56416) antibodies were purchased from Abcam (Cambridge, UK). Anti-p53 (LF-PA20550) and anti-glyceraldehyde 3-phosphate dehydrogenase (GAPDH) (LF-PA41791) antibodies were purchased from Abfrontier (Seoul, Republic of Korea).

### 2.10. Serum Analysis of Klotho, Phosphate, and Mouse Bone Metabolism Proteins

Collected serum samples were stored at −70 °C before batch assay for Klotho levels using MyBioSource Mouse Klotho (KL) ELISA Kit (MBS015591, San Diego, CA, USA), according to the manufacturer’s instructions. Serum phosphate was analyzed using the BioVision phosphate colorimetric assay kit (K410-500, Milpitas, CA, USA) according to the manufacturer’s protocol. Mouse bone metabolism analyses including FGF-23, DKK-1, and sclerostin were performed using MILLIPLEXMAP Mouse Bone Magnetic Bead Panel (MBNMAG-41K, Millipore, Billerica, MA, USA), according to the manufacturer’s protocol.

### 2.11. Statistical Analysis

Results are shown as the mean ± S.E.M. of three experiments. Statistical analyses were performed using the Student’s *t*-test when the data consisted of only two groups (Graphpad Software v7, San Diego, CA, USA). A *p* < 0.05 were considered significant, and a *p* < 0.01 was considered to be highly significant. Quantification of Western blot analysis was performed using the Multi Gauge version 3.0 program (Fujifilm, Tokyo, Japan).

## 3. Results

### 3.1. Characteristics of PMWCNTs and TMWCNTs

**Figure 1:**
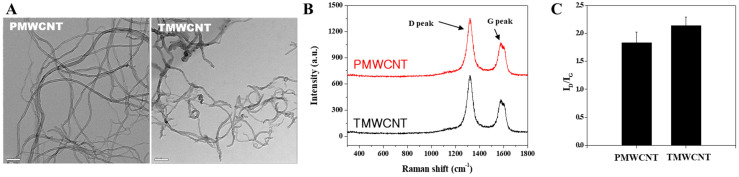
Characteristics of PMWCNTs and TMWCNTs. (**A**) TEM image of PMWCNTs (**Left**) and TMWCNTs (**Right**). (**B**) Raman spectra of PMWCNTs (Red) and TMWCNTs (Black). (**C**) Intensity ratio of Raman D peak and G peak. TMWCNTs show higher I_D_/I_G_ ratio than that of PMWCNTs.

TEM images of PMWCNTs and TMWCNTs shows that the diameter and length of TMWCNTs became smaller and shorter than those of PMWCNTs (Figure 1A). Raman spectra of each MWCNTs displayed two major peaks; the tangential mode or so-called G band at 1601 cm^−1^ and D-band at 1289 cm^−1^ assigned to carbonaceous compounds and defects of MWCNTs (Figure 1B). The intensity ratio of D-band to the G-band (I_D_/I_G_) in TMWCNTs is higher than that of PMWCNTs, meaning that PMWCNTs have less surface defects than TMWCNTs (Figure 1C). These defects can act as bonding sites for better functionalization on the side wall. Inductively coupled plasma atomic emission spectroscopy (ICP-AES) results also shows that by the acid treatment, TMWCNTs contain less metal catalyst remnants compared to PMWCNTs, such as aluminum and ferrite (Table 1).

### 3.2. Dosage Determined Using the Maximum Tolerated Dose (MTD) and Animal Experiments

**Figure 2:**
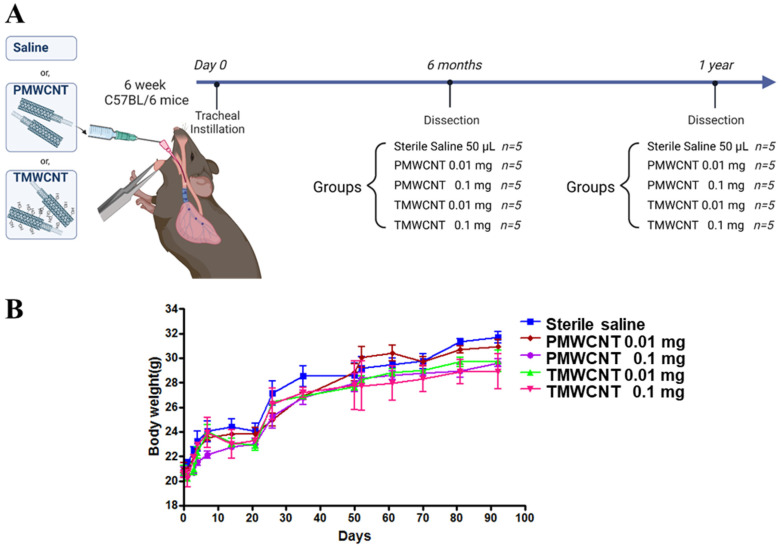
Schematic diagram of the animal experiment and body weight change during 3 months. (**A**) Six-week-old mice were treated with either 50 μL sterile saline, PMWCNTs, or TMWCNTs suspended in sterile saline. Five mice were included in each group, and the concentrations of PMWCNTs and TMWCNTs were either 0.01 mg/50 μL or 0.1 mg/50 μL, with each mouse receiving 50 μL of the respective treatment. The mice were dissected at 6 months and 1 year post-instillation to investigate the long-term effects of the treatments. Created with BioRender.com (**B**) Effects of MWCNTs on mouse body weight gain up until 92 days post-exposure. The average body weight of the control group was 31.7 ± 0.81 g, while the high dosage PMWCNT and TMWCNT groups had an average body weight of 29.59 ± 0.56 g and 28.89 ± 0.83 g each. The average weight of the mice at the start point was approximately 20 g. No toxicity, assessed and defined as a 10% weight loss, was observed up to 0.1 mg/mouse of TMWCNTs and PMWCNTs. Each bar represents the mean ± S.E.M. (n = 5).

The experimental mice received sterile saline or 0.01 mg or 0.1 mg of PMWCNTs or TMWCNTs, as indicated in the schematic diagram, and were dissected at 6 months and 1 year post-instillation (Figure 2A). After a post-exposure period of 92 days, the average body weight of the saline control group was 31.7 ± 0.81 g. In contrast, the group exposed to 0.1 mg of PMWCNTs had an average body weight of 29.59 ± 0.56 g, while the group exposed to 0.1 mg of TMWCNTs had an average body weight of 28.89 ± 0.83 g. The average body weight of the mice at the start of the experiment was approximately 20 g. No toxicity, defined here as a 10% weight loss, was observed up to the dose of 0.1 mg/mouse for both PMWCNTs and TMWCNTs. Therefore, the maximum tolerated dose (MTD) of PMWCNTs and TMWCNTs was established at 0.1 mg/mouse (Figure 2B).

### 3.3. Renal Dysfunction and Histologic Change at 6 Months Post-Instillation of MWCNTs

**Figure 3:**
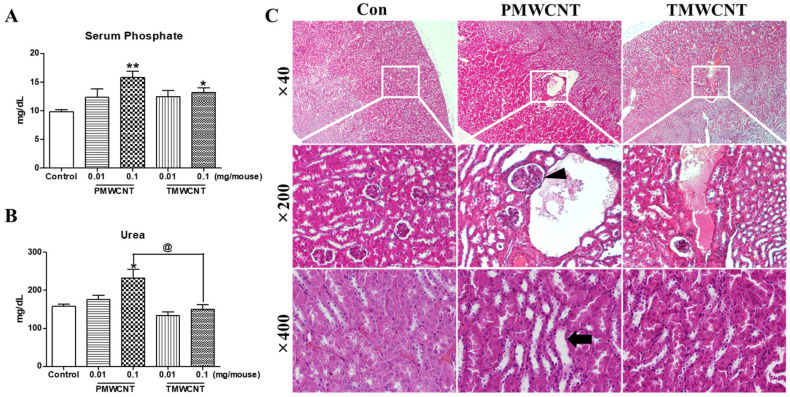
Alteration of serum phosphate, blood urea nitrogen level and kidney histology by MWCNTs after 6 months of instillation. (**A**) Serum phosphate level of mice and (**B**) Serum blood urea nitrogen level of mice after treatment with saline, PMWCNTs, and TMWCNTs. (**C**) H&E staining of sections of the cortex (×200) and medulla (×400) of the kidneys at 6 months post instillation of saline, PMWCNTs, and TMWCNTs. Arrows indicate increased interstitial volume in the PMWCNT-treated group. Arrow heads indicate increased glomerular size. Representative figures of five individuals from each group. Magnifications are indicated in the figures. Error bars indicate mean ± S.E.M. * *p* < 0.05 and ** *p* < 0.001 indicate statistical difference compared to the control group. ^@^ *p* < 0.05 is statistical difference between 0.1 mg of PMWCNTs and TMWCNTs.

At 6 months post instillation, the serum phosphate level was increased significantly in mice treated with 0.1 mg of PMWCNTs and TMWCNTs (Figure 3A). The increment of blood urea nitrogen (BUN) was significant in mice treated with 0.1 mg of PMWCNTs whereas the increase in TMWCNT-treated mice was not significant (Figure 3B). The elevation of BUN in mice treated with 0.01 mg of PMWCNTs was significantly different from that in mice treated with 0.01 mg of TMWCNTs (Figure 3B). In H&E staining of kidney paraffin sections 6 months after PMWCNT and TMWCNT administration, increased glomerular size (arrowhead) and increased interstitial volume (arrow) were observed in the PMWCNT-treated group compared to the saline control and the TMWCNT-treated group (Figure 3C).

### 3.4. Kidney and Serum Klotho Levels of Mice at 6 Months Post Instillation of MWCNTs

**Figure 4:**
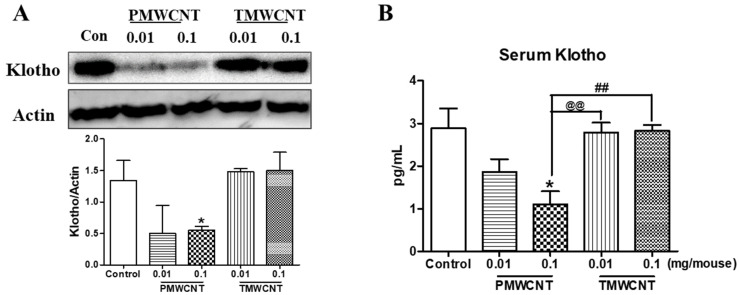
Tissue and serum Klotho level in mice after 6 months instillation. (**A**) Western blot analysis of Klotho in the kidneys of mice after 6 months of treatment with PMWCNTs and TMWCNTs. (**B**) Serum Klotho level of mice exposed to saline, 0.01 mg and 0.1 mg of PMWCNTs and TMWCNTs, respectively. * Statistically different (*p* < 0.05) compared to the control group (n = 5). ^@@^ Statistically different (*p* < 0.01) between the two indicated groups. ^##^ Statistical difference (*p* < 0.01) between the two indicated groups.

After 6 months of instillation, the expression level of kidney klotho was significantly decreased in the 0.1 mg/mouse PMWCNT-treated group according to the results of Western blot analysis, whereas the TMWCNT-treated group was not affected (Figure 4A). The serum Klotho level was significantly reduced in the 0.1 mg/mouse PMWCNT-treated group compared to the control and TMWCNT-treated groups (Figure 4B).

### 3.5. Activated Inflammatory, Fibrotic and Apoptotic Pathways, and Insufficient Autophagy in the Kidneys of 6-Month Post-Tracheal PMWCNT Instillation Mice

**Figure 5:**
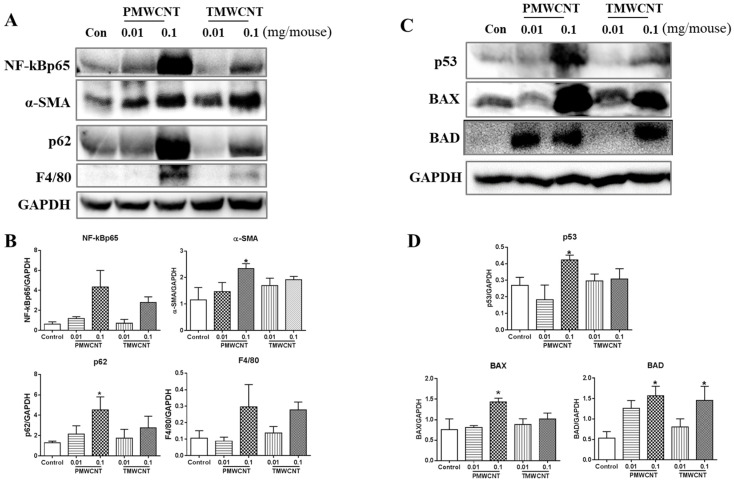
Activated inflammatory, fibrotic and apoptotic pathways, and insufficient autophagy were observed in the kidneys at 6 months post tracheal instillation of PMWCNTs. (**A**) Western blot analysis of NF-κBp65, phospho-smad2, alpha-SMA, p62, F4/80 and MCP1 in the kidney of mice 6 months after the administration of PMWCNTs and TMWCNTs, and their densitometric analysis (**B**). (**C**) Western blot analysis of p53, BAX, and BAD in the kidney of mice after 6 months of treatment with PMWCNTs and TMWCNTs, and their densitometry graph (**D**) (n = 5). The bands of p53, BAX, and BAD were further analyzed using densitometry. The intensity of the p53, BAX, and BAD bands were separated by the intensity of the GAPDH band. Each bar represents the mean ± S.E.M. (n = 5). * Statistically different (*p* < 0.05) compared to the control group (n = 5).

In the kidneys of the mice at 6 months post instillation, the expression level of NF-kBp65 was significantly increased in the 0.1 mg of PMWCNT-instilled group (Figure 5A,B). Abnormal autophagic accumulation was also observed in the 0.1 mg of PMWCNT-treated mice, as shown by a significant increment in p62 expression level (Figure 5A,B). Western blot analysis of alpha-SMA, F4/80 and MCP1 in the kidney of the mice 6 months after the administration of PMWCNTs and TMWCNTs showed a dose-dependent increase, with the highest expression level in the mice treated with 0.1 mg of PMWCNTs (Figure 5A,B). The expression levels of pro-apoptosis proteins were also increased significantly in the kidney of the mice treated with 0.1 mg of PMWCNTs (Figure 5C,D). The expression levels of p53 and BAX were increased and BAD was significantly increased in the 0.1 mg of PMWCNT-treated mice (Figure 5C,D).

### 3.6. Histologic Change of Kidney after 1 Year of MWCNTs Instillation

**Figure 6:**
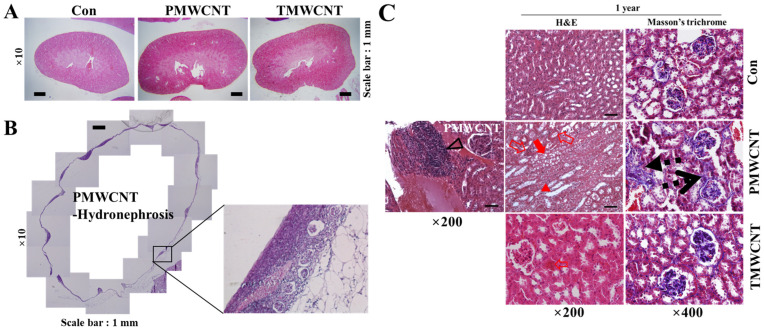
The effects of administering multi-walled carbon nanotubes (MWCNTs) on the morphology of mouse kidneys after one year. (**A**) Hematoxylin and eosin-stained images show the morphologies of mouse kidneys treated with saline, PMWCNTs, and TMWCNTs. (**B**) PMWCNTs induce hydronephrosis after one year. Inset shows traces of the glomerulus. (**C**) Paraffin-embedded kidney tissue sections one year after the administration of PMWCNTs and TMWCNTs were stained with H&E and Masson’s trichrome. Red arrow indicates tubular epithelial vacuolation. Empty arrow indicates tubular epithelial necrosis. Arrow head indicates tubular dilation. Empty arrow head indicates lymphocyte infiltration. Dashed arrow and dashed empty arrow indicate tubulointerstitial fibrosis and glomerulosclerosis, respectively. Magnification and scale bar are indicated in each figure.

At 1 year post instillation, the overall morphology of the kidneys showed empty spaces in the mice treated with 0.1 mg of PMWCNT per mouse, while the control and TMWCNT groups did not show abnormal morphology (Figure 6A). Hydronephrosis was observed in the 0.1 mg of PMWCNT-administered mice and traces of the glomerulus were observed in the remaining cortex (Figure 6B). Compared to the control and TMWCNT groups, H&E staining of the PMWCNT-treated group showed increased tubular epithelial vacuolation (Arrow), tubular epithelial necrosis (Empty arrow), tubular dilation (Arrow head), and lymphocyte infiltration (Empty arrow) (Figure 6C). In addition to this, interstitial fibrosis (Dashed arrow) and glomerulosclerosis (Dashed empty arrow) were observed in the mice treated with 0.1 mg of PMWCNTs on Masson’s trichrome staining (Figure 6C). These pathological observations are summarized in Table 2. PMWCNTs caused significant tubular degeneration and lymphocyte infiltration compared to the TMWCNT-treated group (Table 2).

### 3.7. Changes in Serum Indicators Related to Kidney Function and Aging

**Figure 7:**
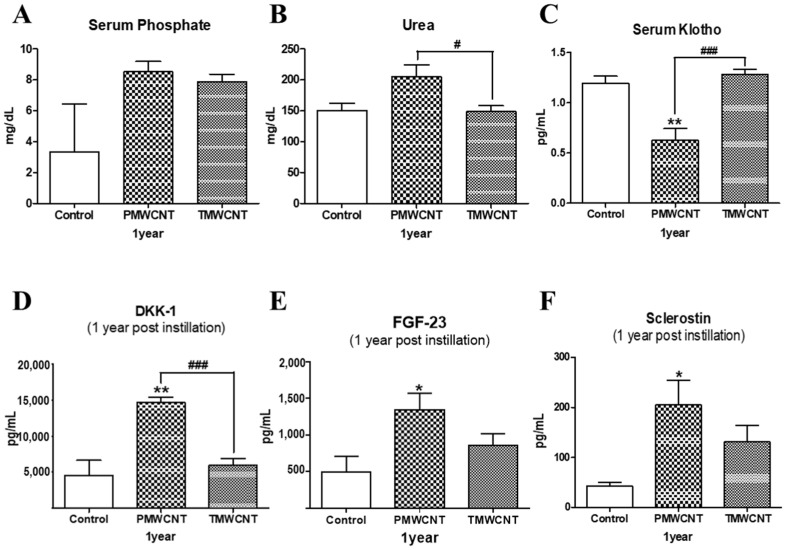
Serum analysis of mice after 1 year of instillation. (**A**) Serum phosphate, (**B**) Blood urea nitrogen, (**C**) Serum Klotho, (**D**) Serum DKK-1, (**E**) Serum FGF-23 and (**F**) Serum sclerostin levels after 1 year of treatment with saline, PMWCNTs, and TMWCNTs. Each bar represents the mean ± S.E.M. (n = 5). * Statistically different (*p* < 0.05) compared to the control group. ** Statistically different (*p* < 0.01) compared to the control group. ^#^ Statistical difference (*p* < 0.05) between the two indicated groups. ^###^ Statistically different (*p* < 0.001) between the two indicated groups.

On serum analysis of mice after 1 year of instillation, phosphate levels were increased in the PMWCNT- and TMWCNT-treated groups (Figure 7A) and blood urea nitrogen levels were significantly increased in the PMWCNT-treated group, compared to the TMWCNT-treated group (Figure 7B). Serum Klotho levels were significantly decreased in the PMWCNT-treated group, compared to the control and TMWCNT-treated groups (Figure 7C). Serum DKK-1 (Figure 7D), FGF-23 (Figure 7E), and Sclerostin (Figure 7F) levels in the PMWCNT-treated group were significantly increased compared to the control.

## 4. Discussion

Abnormal kidney function is a common issue that promotes premature aging and cellular senescence [12,32]. This effect is related to several mechanisms, including increased reactive oxygen species [18], mitochondrial damage [19], persistent inflammation [20], phosphate toxicity [21], and decreased Klotho expression [11]. Since lung exposure to MWCNTs is reported to generate free radicals and persistent inflammation [3,33], here, we investigated the possible toxic effects of MWCNTs on the kidneys and premature aging. Our study shows that intratracheal exposure to MWCNTs can induce kidney inflammation and related premature aging phenotypes. By comparing the effect of PMWCNTs and TMWCNTs, we also discovered that the effects were different in that PMWCNT-induced kidney aging more than TMWCNTs. The determination of premature kidney aging was largely achieved using pathological features of the kidneys (Table 2, Figure 3 and Figure 6), specifically, decreased levels of serum Klotho (Figure 4B and Figure 7C), increased serum FGF-23 (Figure 7E), and increased apoptosis and inflammation (Figure 5) in the kidneys.

The aging kidney exhibits various glomerular and tubulo-interstitial changes [25]. We observed several structural changes in the kidneys of mice treated with 0.1 mg of PMWCNTs. After 6 months of exposure, glomerular size increase and increased interstitial volume was observed; after 1 year, tubular epithelial vacuolation, tubular epithelial necrosis, tubular dilation, lymphocyte infiltration, interstitial fibrosis, and glomerulosclerosis were apparent (Figure 3 and Figure 6). These are all typical hallmarks of kidney aging [34,35]. In particular, one year after the administration of 0.1 mg of PMWCNTs, two of the five mice exhibited acquired unilateral hydronephrosis (Figure 6B), which is a naturally occurring disease that impairs free urine flow. Many etiologies leading to hydronephrosis are of particular significance to aging mice [36]. Since alterations in tubular function accompany anatomical involvement, tubular scarring leads to reduced Na-K ATPase, urea transporter, and proton pump in the elderly, resulting in electrolyte and mineral imbalances [37].

Next, we conducted serum analysis, and elevated serum phosphate (Figure 3A and Figure 7A) and blood urea nitrogen (Figure 3B and Figure 7B) levels were observed. These have been reported to be responsible for accelerating premature aging and causing decreased kidney function, respectively [21,24]. Increased serum phosphate level is reported to be universal in patients with advanced CKD or end-stage renal disease (ESRD) [16], and ESRD patients are known to show aging-like phenotypes including vascular calcification and metabolic bone disease [12] induced by decreased Klotho expression in the kidneys [38] as well as increased serum FGF-23 levels [39]. The reduced kidney and serum Klotho levels (Figure 4 and Figure 7C) in addition to the increased FGF-23 (Figure 7E) found in our study also coincide with these reports. Klotho, an anti-aging protein expressed in the distal convoluted tubules [23], is reported to induce hyperphosphatemia as its inhibitory effect on the proximal tubule Na-coupled phosphate transporter is lost [40]. Since phosphate is thought to be a major toxicological molecule that induces premature aging-like phenotypes [21] and genotoxic stress [41], the hyperphosphatemia shown in this study supports the theory of possible premature effects from PMWCNTs and TMWCNTs. In addition to this, decreased Klotho is known to be associated with increased susceptibility to oxidative stress via the activation of the insulin growth factor-1(IGF-1) pathway [22]. Emerging evidence also suggests that Klotho, itself, may be directly involved in the regulation of cellular senescence [42,43]. Increased circulating FGF-23 (Figure 7E) has also been reported as a consequence of decreasing nephron numbers with age, progression of CKD [39,44], and loss of the integrity of the Klotho signaling pathway, which accompanies mild hypercalcemia, vascular calcification, and elevated 1,25(OH)_2_ vitamin D_3_ [45,46]. FGF23 suppresses Cyp27b1, a gene that encodes 25-hydroxy-vitamin D 1α-hydroxylase, an enzyme that is essential for 1,25(OH)_2_ vitamin D_3_ synthesis in the kidneys [47]. Therefore, perturbation of the klotho-FGF-23 endocrine axes, which accompanies hyperphosphatemia, appears to be a major reason for the premature aging effects from PMWCNTs observed in this study.

This study also demonstrated an increased aging phenotype such as inflammatory response, abnormal autophagy, and increased apoptosis in the kidneys (Figure 5). Chronic inflammation, which is characterized by a progressive accumulation of macrophages, shown here as F4/80 increments (Figure 5A), and lymphocytes in the kidney interstitium, may induce or potentiate renal aging [13]. The major mechanisms whereby chronic inflammation may promote kidney aging include the induction of fibrosis by inflammatory cells through pro-fibrotic cytokines [48] and the enhancement of cell apoptosis [49]. Recent studies have revealed the renoprotective roles of autophagy against aging both in proximal tubular cells and podocytes [50]. Abnormal p62 accumulation in MWCNT-treated mice results in an impaired ability to induce autophagy and the aging phenotype (Figure 5A).

We further investigated whether PMWCNT-induced kidney damage could affect bone mineral metabolism and vascular calcification, which are major categories of aging, using related serum proteins including DKK-1, FGF-23, and sclerostin (Figure 7D,F). In chronic kidney disease, mineral and bone disorder is an extremely serious complication [17], and a disruption in bone and mineral homeostasis can increase cardiovascular mortality by inducing atherosclerosis-stimulated arterial calcification [51]. The serum proteins sclerostin and DKK-1 are soluble inhibitors of canonical Wnt signaling and have been identified as components of parathyroid hormone signal transduction [52]. Mounting evidence indicates that the increment of these proteins may be related to renal osteodystrophy correlated with aging [53]. We found a significant increase in sclerostin and DKK-1 in the PMWCNT-instilled group, supporting the theory that PMWCNTs can initiate chronic kidney disease-associated bone and mineral disorder through the inhibition of Wnt signaling. However, studies exploring the clinical correlations of serum DKK1 and sclerostin levels have so far yielded conflicting results regarding bone disorders [53,54]. Biological variability and analytical issues account at least partly for this inconsistency. Interestingly, a recent report by Weitzmann et al. indicated that bioactive silica nanoparticles can reverse age-associated bone loss in aged mice, and they showed increased serum osteocalcin levels and bone density in the bioactive silica-treated group [55]. Although the tested materials, concentrations, and experimental conditions are totally different, and even the results look conflicting at first glance, our results in part coincide with the results of Weitzmann et al., in that there is a report arguing that reduced Klotho levels is related to increased tibial trabecular bone density since Klotho has the opposite effect on Wnt signaling [56]. Serum FGF23 is reported to be independently correlated with vascular calcification but not mineral density in CKD patients [57] while vascular calcification and osteoporosis appear to be independent processes in elderly women [58].

There are possible explanations for the reasons of the different degree of toxicity of P PMWCNTs and TMWCNTs in kidney. Through acid treatment, TMWCNTs are more hydrophilic than PMWCNTs because of surface functional groups on them and consequently more dispersible (Figure 1). Our result coincides with the report by Sayes et al., [59] that as the degree of sidewall functionalization increases, the SWCNT becomes less cytotoxic. Another study also presented that suspended CNT-bundles were less cytotoxic than asbestos, while rope-like agglomerates induced more severe cytotoxic effects than asbestos fibers at the same concentrations [60]. Not just an in vitro study, also an in vivo study showed that MWCNTs with higher degree of agglomeration are more toxic, in terms of their prolonged persistence in the body [61]. Agglomerated MWCNTs retained in the lungs and liver and were not eliminated completely in 28 days, while the well-dispersed ones remained as fewer aggregates in the lungs and liver, and be easily eliminated [61]. Our previous study also supports that aggregated PMWCNTs cleared more slowly than TMWCNTs [2]. Persistently accumulated agglomerated MWCNTs in the lungs caused consistence systemic inflammatory responses, and this might be the major reason for the differential toxic effect of PMWCNTs and TMWCNTs on the kidneys. Another possible reason is that PMWCNTs contain a higher amount of iron and aluminum compared to TMWCNTs. It has been shown that iron can cause kidney toxicity by inducing oxidative stress, mitochondrial dysfunction, inflammation, and cell death [62]. Aluminum toxicity is also observed when plasma levels of aluminum exceed 0.2 μg/mL, leading to inhibition of enzyme activity and protein synthesis, alterations in nucleic acid function, and changes in cell membrane permeability [63]. Altogether, these suggest that synthesizing dispersible suspension is one of the key requirements for safe usage of MWCNTs in the applicable field.

In this study, we could not directly evaluate bone mineral density because we did not expect the possibility of kidney toxicity from PMWCNT and TMWCNT exposure through the lung after one year post administration, so we did not preserve the bone as an analytic sample. Moreover, functional analysis of the kidney’s glomerular filtration rate (GFR) could be a potential strength that strengthens the conclusions of this study. Although our study warrants further experiments, an intensified focus for the evaluation of factors that dictate the production as well as the release of humoral factors from the bone that can induce pathological changes at distant sites could open a new avenue for evaluating the parameters of nanotoxicity for possible aging effects.

## 5. Conclusions

Our study demonstrates that PMWCNT exposure through the lungs can induce chronic kidney damage associated with premature aging within 1 year of instillation, evidenced by histological changes, increased inflammation, abnormal autophagic accumulation, and increased apoptosis in the kidneys. In addition to this, increased serum phosphate, decreased serum Klotho, and increased bone mineral metabolism-related hormones including DKK-1, FGF-23, and Sclerostin also indicate kidney-related premature aging in MWCNT-treated mice. Furthermore, by comparing PMWCNTs and TMWCNTs, our study shows that acid-treatment can mitigate the kidney effects of MWCNTs. Although our study cannot explain whether premature aging or kidney damage comes first, and limited number of mice were used, our findings can contribute to better understanding of the premature aging effect from lung exposure to MWCNTs on the kidneys.

## Figures and Tables

**Table 1:** The differences in metal catalyst contents between PMWCNTs and TMWCNTs.

	PMWCNT (ppm)	TMWCNT (ppm)
Mn	nd	nd
Co	nd	nd
Ni	nd	nd
Cu	nd	nd
Zn	nd	8.96
Al	8973.5	613.28
Fe	12,018.0	1082.64
Ti	nd	nd
Pt	nd	nd

nd: not detected.

**Table 2:** Summary of pathological observations indicative of PMWCNT- and TMWCNT-induced renal dysfunction.

	Con	PMWCNT	TMWCNT
**Tubular injury**			
Tubular degeneration	0.21 ± 0.021	1.833 ± 0.167 *	0.900 ± 0.233
Mononuclear/lymphocytic infiltrates	0.00 ± 0.00	0.833 ± 0.401 *	0.32 ± 0.100

Grade 0: None. Grade 1: Mild. Grade 2: Severe. * indicates *p* < 0.05, *t*-test between PMWCNTs and TMWCNTs.

## Abbreviations

PMWCNT: pristine multi-walled carbon nanotube, TMWCNT: Acid-treated multi-walled carbon nanotube, MTD: Maximum tolerated dose, NF-κBp65: anti-nuclear factor kappa-light-chain-enhancer of activated B cells p65, α-SMA: alpha-smooth muscle actin, GAPDH: glyceraldehyde 3-phosphate dehydrogenase, DKK-1: Dickkopf-related protein 1, FGF-23: Fibroblast growth factor 23, BUN: Blood urea nitrogen, BAD: Bcl-2-associated death promoter, BAX: Bcl-2-associated X protein.
